# Association of high-sensitivity cardiac troponin T concentrations with the left ventricular global function index after ST-segment elevation myocardial infarction

**DOI:** 10.1186/1532-429X-17-S1-P134

**Published:** 2015-02-03

**Authors:** Hans-Josef Feistritzer, Sebastian J Reinstadler, Gert Klug, Marie-Therese Gröber, Johannes M Mair, Michael Schocke, Wolfgang-Michael Franz, Bernhard Metzler

**Affiliations:** University Clinic of Internal Medicine III, Cardiology and Angiology, Medical University of Innsbruck, Innsbruck, Austria; Departement of Radiology I, Medical University of Innsbruck, Innsbruck, Austria

## Background

The left ventricular global function index (LVGFI) is a novel indicator of cardiac performance. Recently, the LVGFI was linked to left ventricular ejection fraction and infarct characteristics in patients presenting with acute ST-segment elevation myocardial infarction (STEMI). In this study we sought to investigate the association of serially measured high-sensitivity cardiac troponin T (hs-TnT) concentrations and LVGFI assessed early after acute STEMI.

## Methods

122 patients with first STEMI (mean age 58 ± 11 years) reperfused by primary percutaneous coronary intervention (p-PCI) were enrolled in this observational study. All patients underwent cardiac magnetic resonance (CMR) imaging within the first week after STEMI. Left ventricular dimensions and function were measured by cine true-FISP sequences. hs-TnT concentrations were assessed serially for up to 48 hours after p-PCI by a commercially available immunoassay. Fisher's r to z transformation was applied for comparison of correlation coefficients.

## Results

The median delay to reperfusion was 205 min (IQR 144 to 391 min). The mean LVGFI was 35 ± 8 %. The median value of maximum hs-TnT concentrations was 5043 ng/l (IQR 2322 to 8104 ng/l). The LVGFI was significantly associated with maximum creatine kinase activity (r = -0.473, p < 0.001) and with maximum hs-TnT concentration (r = -0.478, p < 0.001). Statistically significant correlations were also detected between LVGFI and single time-point hs-TnT concentrations (all r < -0.422, all p < 0.001). Receiver operator characteristics analysis (area under the curve: 0.85, 95% CI 0.75 to 0.95) revealed hs-TnT concentrations measured 6 hours after p-PCI to most accurately predict a LVGFI below the median value of 34 %.

## Conclusions

This study demonstrates that hs-TnT concentrations assessed serially after acute STEMI are associated with LVGFI. hs-TnT levels measured 6 hours after p-PCI best predicted a LVGFI below the median value.

## Funding

Austrian Society of Cardiology.

